# Population dynamics and demography of Covid-19. Introduction

**DOI:** 10.1186/s41118-021-00143-5

**Published:** 2021-12-16

**Authors:** Viviana Egidi, Piero Manfredi

**Affiliations:** 1 Dipartimento di Scienze Statistiche, Sapienza Università di Roma, Piazzale Aldo Moro, Rome, Italy; 2 Dipartimento di Economia e Management, Università di Pisa, Via Ridolfi 10, Pisa, Italy

**Keywords:** COVID-19 pandemic, Mitigation measures, Direct health impact vs societal impact, Demography, Modern science, Pandemic risk

## Introduction

Since its early debut in Wuhan, China, during winter 2019, the COVID-19 pandemic has devastated human communities all over the world as a global hurricane (Horton, 2021; Lupton & Willis, 2021). Its dramatic impacts on health, on the population, on the economy and on the society as a whole have suddenly put us in touch with tragedies, as the 1918 Spanish flu pandemic and the Great 1929 recession, that we were used to consider as belonging to our history only, especially in western countries (Livi-Bacci, 2017, 2021).

Unlike the aforementioned past tragedies, the COVID-19 pandemic has been the first one in mankind history under the lenses of science, being thus entirely monitored, described, and communicated daily to the population, by the tools of statistics. Since the beginning of the story, mass media—from newspapers to newscasts to the internet—have been permanently occupied by experts talking about data, graphs and tables communicating to us the daily news from the pandemic battlefield. This certainly had the merit to enable citizens and the entire public opinion to finally familiarize in their daily life with science and its tools, though it also led to somewhat undesired consequences, such as the parallel “infodemia” and related information disorder (Bursztyn et al., 2020; Cinelli et al., 2020). Overall, however, this ubiquitous presence and monitoring by science was not able to avoid that the eventual—direct and indirect—impact of the pandemic, and of its mitigation measures, was catastrophic (Horton, 2021; Lupton & Willis, 2021) and will likely last for a long time in the future.

In this nasty scenario, an indisputable merit of the continued focus of science on the pandemic lied in the endless list of scientific questions that were posed and in the resulting unprecedented mobilization of scientific interests and resources. For example, a search on PubMed repository at current date (October 18th, 2021) revealed 187,837 indexed scientific papers including the word “COVID-19” (PubMed 2021). Obviously, many of the raised questions are still un-answered but their investigation is opening formidable tasks for research in almost all scientific fields, including demography. With hindsight, most pre-COVID-19 research on topics as pandemic risk, pandemic preparedness, and especially that of societal protection from such events, was largely confined to a restricted number of specific fields mainly related to the areas of public health and medicine, and their statistical and mathematical modelling. Indeed, on a one hand, a wide and high-quality literature emerged in public-health pandemic preparedness during the fight against pre-COVID alerts such as the HIV-AIDS epidemic, the 2003 SARS outbreak due to the SARS-COV-1 virus, the avian-flu alert, the 2009 H1N1 “mild” pandemic, the *Middle East Respiratory Syndrome* (MERS) crisis, the repeated devastating large-scale Ebola epidemics in Central-Africa and the Zika alert (Cooper et al., 2006; Ferguson et al., 2005, 2006; Fraser et al., 2009; Merler et al., 2015; Riley et al., 2003; Zhang et al., 2017). This past research has put the conceptual and statistical substrate that has been used during the COVID-19 pandemic to support governmental decisions. However, on the other hand, entire disciplinary areas remained—with a few exceptions—immune to this call for research, despite the evidence that the impact of a pandemic attack would have gone largely beyond the medical and public health domains. This phenomenon is somewhat surprising if one recalls that communicable diseases still represented—*long before the COVID-19 pandemic*—the major component of mortality in a large part of the developing world, especially in Sub-Saharan Africa in view of the persistence of major killers such as malaria, tuberculosis, HIV and other diarrheal and respiratory diseases (Bloom & Canning, 2004; Bloom et al., 2018; Murray et al., 2015). The explanation of this lack of interest by many disciplinary areas possibly lies in the western “oblivion” (Gori et al., 2020) that followed from the dramatic levels of infectious diseases control achieved by western populations along the mortality and epidemiological transitions (Livi-Bacci, 2017; Murray et al., 2015; Snowden, 2019; Solomon & Murray, 2002) as well as through the widespread prevention allowed by mass vaccination since the 1950s (e.g., van Wijhe et al. 2016), that made most traditional communicable infections extremely rare in these populations. The current pandemic has, finally, upturned this state of affairs, impressing a formidable new momentum to the investigation of communicable diseases from a broader societal standpoint.

Therefore, trusting that—thanks to widespread COVID-19 vaccination—we are getting out of the worst, it should be finally arrived the right moment for starting to appropriately answer the many un-answered questions and to—hopefully—transforming this ugly tragedy into a great lesson-to-be-learned by science and policy for the future battles on mankind.

In relation to what we stated before, as demographers, we should finally be in the position to characterise the conditions and the contexts that have contributed to favour the spread of the epidemic and the impact of its most severe consequences and, particularly, to assess the quality of the tools that have been used to evaluate the impact of the virus on mortality. This will be key to start understanding the consequences—at both the individual and the societal level—due to the pandemic and its mitigation measures.

At the time we were first writing the call for this Genus Thematic series in early Spring 2020, the COVID-19 first wave was just ongoing (and we were preconizing that it would not remain the only one). Nonetheless, some of its main features and keywords, such as *complexity*, *multi-dimensionality* of effects, and *pervasiveness*, were already manifest from the multiplicity of involved risks of both direct and indirect nature resulting not only from the disease per se but also, and sometimes primarily, by the enacted mitigation measures.

The above keywords (multidimensionality, complexity and pervasiveness) are well mirrored by the heterogeneity of the subjects investigated in the eight manuscripts that were eventually selected to be included in this Thematic Series. Among these, two (Soneji et al., 2021; Vanella et al., 2021) have been dealing with the chief demographic topic in relation to any pandemic crisis, namely the magnitude, timing and structure of mortality (Dowd et al., 2020; Goldstein & Lee, 2020). Three other manuscripts (Bernardi et al, 2021; Giorgi & Boertien, 2021; Makinde et al., 2021) share—though with different perspectives and methodologies—a common underlying theme, namely the conditions—at the individual and collective level—that could either favouring or limiting the spread of a deadly virus as COVID-19, as well as the impact of its direct consequences on health, from serious disease to mortality. These conditions have been identified either in the household structures and housing conditions (the contribution by Makinde et al., 2021), or in population structures (the heterogeneity in co-residence structures analysed by Giorgi & Boertien, 2021) or, finally, in the devastating role played by living in nursing homes especially during the first pandemic wave (Bernardi et al., 2021). One contribution has been more generally dealing with the conditions favouring or hindering social resilience against the multiple risk factors, both direct and indirect, emerged during the pandemic, such as the disruption of social and economic activities and relationship. These were identified in typically social structures, namely the “protection social networks” analysed by Furfaro et al. (2021). Finally, the remaining two manuscripts have investigated the impact of the first phases of the pandemic (and related control measures) on life histories, namely on the transition to adulthood (analysed by Luppi et al, 2021) and on mental and psychological health of university students (Busetta et al, 2021).

Before introducing to the articles included in this Thematic series, a preliminary remark on the pandemic chronology and the timing of this special issue might be useful. As it also happened for many other special issues on COVID-19, the present one was motivated by—and indeed its contents cover—a precise epoch of the pandemic, primarily the first wave or a part of the second one. Clearly, the history of the pandemic was so complex, with so many new events, evidences and discoveries occurred after the first wave, that many of the stories related to the early pandemic epoch might appear to many observers as belonging to a far past. For this reason, and in order to avoid that the published papers appeared excessively "time-contextualized", contributing authors were asked to upgrade their manuscripts in order to set them within the broader context of the COVID-19 pandemic so as to avoid a rapid decay of the interest for the involved topics, analyses and results.

## The thematic series “Population dynamics and demography of COVID-19”

As for the first vein previously mentioned, in an earlier phase of the pandemic much of the public debate has been dealing with the magnitude of COVID-19 mortality. This had been motivated by the difficulties of the routinely used epidemiological measure, namely the case-fatality ratio (CFR). In the case of COVID-19, the difficulties of the CFR involve both the numerator (COVID-19 deaths), due to the difficulties in ascertaining COVID as the principal cause of death, as well as the denominator (COVID-19 confirmed incidence) due e.g., to the large proportion of asymptomatic cases. These difficulties were dramatically magnified during the hot phase of the first pandemic wave (WHO, 2021b). Owing to these difficulties, Soneji et al (2021) relied on available figures of confirmed deaths with an underlying COVID-19 diagnosis in 13 European countries, the US and Canada during the first and a broad part of the second pandemic wave (till February 2021), to compute age-standardised COVID-19 death rates (ASDR). ASDRs were then used to compare COVID-related mortality amongst the cited countries and also within-country for the two cases of Germany and the US. They additionally made a number of mortality-by-cause comparisons between the COVID-related crude death rate (CDR) and the CDRs for a selection of leading causes of deaths. Their main results were that (i) COVID-19 rapidly emerged as the second leading death cause in England & Wales and France (behind cancer) and the third leading cause of death in eleven countries amongst the remaining thirteen, and that (ii) COVID-19 ASDRs showed ample variation within countries, as it is expected due to the heterogeneity in the epidemic state at the moment mitigation measures were actually introduced. Overall, the issue of assessing mortality during an acute pandemic phase is a serious one, from which we should draw useful prescriptions in view of the preparedness towards possible future pandemic events. In the case of COVID-19, this was also made complicated by the lack of an agreed definition of a “COVID-19 death” and of harmonized procedure of data collection amongst the various countries and—further—by the dramatic under-notification in sites severely hit by the pandemic. These difficulties were extensively discussed by the authors.

The limitations and potential lack of reliability of directly measuring COVID-19 mortality from the count of deaths with a COVID-19 diagnosis, has rapidly led—already during the first pandemic wave—to the idea that excess mortality represented instead the most appropriate indicator of the pandemic impact on mortality. In their study, Vanella and coauthors (Vanella et al., 2021) extended Lee-Carter methodology (Lee & Carter, 1992) to estimate COVID-19 weekly age-sex-specific mortality data in 19 European countries by accounting for the numerous statistical issues that are involved, namely autocorrelations between time series, between age-sex groups, and between countries. The authors also claim the importance of their approach in its ability to include comparison windows of any arbitrary lengths. Two points are worth mentioning in relation to their work. The first one is that the concept of excess mortality is by itself a complicated one including an entire range of factors directly and indirectly contributing to it, including (IMHE, 2021): (i) delayed health care during the pandemic; (ii) increases in mental health disorders; (iii) decreases in injuries because of general reductions in mobility associated with social distancing mandates during strong mitigation phases; (iv) reduced transmission of other viruses, most notably influenza, due to social distancing measures; (v) reductions in mortality due to the harvest effect on frail individuals. The second point is a more methodological one, related to the optimal choice of the comparison time-window against which the excess mortality is assessed. The authors are quite optimistic in the power of their methodology allowing to include any desired length and therefore emphasize the merits of including as much past information as possible. However, this is an important open issue: the assessment of excess mortality depends on the past dynamical trajectories of mortality, which can be disrupted by events or occasional mortality trends that could alter excess assessments in an inappropriate way and that can differ among countries.

Among the conditions and behaviours that may affect risk factors at the individual level, household composition, the degree of its isolation and its sanitation capacities are relevant factors in the process of transmission and control of many infectious diseases, playing an important role both on the force of infection and on the outcome of the disease. Makinde and coauthors in this Series (Makinde et al., 2021) used DHS data to explore household conditions which can facilitate or contrast the transmission of the SARS-CoV-2 virus. Their focus is on sub-Saharan African countries, where the major pathway to controlling infection transmission and its worst consequences still rests almost exclusively on sanitation practices and social distancing (Bloom & Canning, 2004; Murray et al., 2015). Two variables are used as indicators of the prevention capacity: handwashing and self-isolation abilities. Additionally, the proportion of household’s members aged 60 and over is used as an indicator of the potential risk of dying when the infection has entered the household. Controlling for the main socioeconomic variables, both at the household and the contextual level, strong relationships were found between the two risk factors indicating the tendency to create situations in which disadvantages cumulate. Poor households have lower prevention capacities and the risk of the elderly living in these households being infected is the highest. The same is true for the negative outcome of the infection that is influenced by the same poor socio-economic condition producing higher incidence. A main conclusion is that factors influencing COVID-19 incidence and mortality are complex and context-dependent, so that effective strategies aiming to prevent epidemic spread can be hardly generalized to all sub-Saharan countries but must take into account for their specificities.

A central point in relation to the generalized national lockdowns that were decreed in many countries worldwide during the first pandemic wave, was that lockdowns had a marked "switching" effects in terms of groups, setting and activities at risks of being hit by the infection and its serious consequences. In particular, following the general population confinement enacted by lockdowns, households have become a special at-risk setting (Leng et al., 2020; Roberton & Carter, 2020), due to the dramatic increase in time spent at home jointly with the difficulties to keep severe protections rules in a setting—the household—where contacts are unavoidably more intimate than in other ones. In their work, Giorgi and Boertien (2021) used a micro-simulation approach informed by census data for France to assess the extent to which the heterogeneity in French co-residence structures might interact with socio-economic inequalities in shaping pandemic mortality. The analysis suggests that among the older age groups, those actually most hit by the pandemic, the most vulnerable are represented by households headed either by a highly educated or a native-born subject, despite the fact that large households are more common instead among the lower educated groups or among the foreign born. This seemingly paradoxical result is well explained by the existing socio-economic gradient that makes higher educated people to be still living with their partners at higher ages, thereby potentially causing higher mortality attack rates.

Among the many scaring happenings of the first pandemic wave, a major one was represented by the catastrophe occurred in nursing homes, suggesting that living in such settings represented, other things being equal, a critical factor of increased risk of infection and mortality. This phenomenon was particularly dramatic in Italy (ISS, 2020). The nature of nursing homes as large households composed by highly frail individuals interconnected by either social activities, as meals, or by a common (and highly mobile) nursing staff (another category especially at risk of acquiring the infection during the pandemic), makes them high at-risk environments in that once the pathogen has entered them, very large attack rates can be expected. The contribution by Bernardi et al. (2021) aimed at characterizing the determinants of the probability of becoming a resident of a nursing home in 12 European countries using SHARE data. With all due caveats related to underlying uncertainty, they found that individuals more likely at risk of living in a nursing home are those with a lower educational grade and a household income below the national median. This provides evidence that the COVID-19 pandemic has hit stronger the more economically fragile groups, thereby potentially enhancing the contribution of socio-economic inequality to mortality differences. Nursing homes should definitely become a primary target of future preparedness activities towards new potential COVID-19 waves (e.g., due to new variants) or towards future pandemics.

Some studies in this Thematic Series were rather interested in the consequences of the pandemic and of the subsequent mitigation measures introduced. Indeed, since the virus was a fully new one when it first appeared in Wuhan, with a lack of vaccines and of adequate therapies, the type of interventions enacted during the first dramatic wave could only be very coarse, as exemplified by generalised lockdowns. Generalised lockdowns primarily targeted the first of the three key factors fueling the spread of a virus i.e., the daily number of social contacts. Indeed, given the emergency situation, targeting the other two factors i.e., the duration of the infective phase (through isolation) and the virus contagiousness (through individual protection devices), was rather more complicated in view of the high proportion of pre-symptomatic and asymptomatic transmissions, and of the unclear effectiveness of individual protections (as masks), respectively.

In relation to this, the contribution by Furfaro et al. (2021) focused on the conditions which may increase the adaptive capacity of individuals or, conversely, amplify the negative impact they have suffered because of the social distancing measures. Their work focused on Italy, the second country worldwide after China in facing—from a chronologic standpoint—the arrival of the pandemic tsunami, and considered two target populations: young adults living alone or in a couple (with or without children) and elderly persons (without cohabiting children); both, younger and older persons, living with no other family or non-family members. A social network analysis is used to identify types of social relations and conditions that may exacerbate the impact of social distancing measures. The paper proposes two kinds of ego-centered networks with the goal to better identify the characteristics of individuals most exposed to relational vulnerability among the young adults and the elderly persons: the “easy-to-reach” and the “accustomed-to-reach” network. Additionally, focus is put on the critical situation of persons more exposed to relational vulnerability. They are single persons, both among young adults and the elderly people who declared no other persons to whom they could address in time of serious need. These “isolated” persons represent about the 10% among both young adults and the elderly, with a peak just below 15% among the single elderly men. The latter figures become even higher when considering the accustomed-to-reach network which include people to whom the elderly person has the habit of meeting and spending time together. This condition of isolation, in addition to representing a greater risk of a more negative impact of social distancing measures, also increases the risk of living in a nursing home, where during the most acute phases of the pandemic the mortality risk was the highest, as also considered in the cited contribution by Bernardi et al. (2021) in this Thematic series.

Two papers in this Series addressed the consequences of the pandemic in a highly vulnerable phase of life history, that of youth, when important choices are made, such as leaving the parental home to start an independent life—a theme that has grown dramatically after Billari’s seminal work (Billari, 2004)—or when the education process is to be completed. In their manuscript, Luppi et al. (2021) have assessed the impact of the COVID-19 pandemic and of its mitigation measures on the project of leaving the parental home by young adults in five European countries (Italy, Germany, France, Spain and UK). Requiring some economic independence from the parents, the decision of leaving the parental home, was negatively impacted during the economic crisis resulting from the pandemic especially in those countries where young people have a more vulnerable position in the labour market. Indeed, in countries where young persons are more frequently employed in temporary and less protected jobs or experience a higher unemployment risk, they more frequently postponed or abandoned their projects of leaving parental home as a consequence of the COVID-19 pandemic. Perceived future conditions of the labour market and income also played a significant role in the decision-making process, as well as the general context in which young people live. Young people living in Italy, Spain and UK, where they most frequently have temporary jobs and feel more insecure about the future financial situation, showed the higher probability of negatively revising their projects, compared to France and Germany. As the authors highlight, the institutional framework matters when relevant decisions have to be taken, with familistic and liberal welfare regimes not offering sufficient guarantees to support life plans during times of crisis.

The young and the more educated persons proved to be particularly sensitive to the negative consequences of crisis periods. The article by Busetta et al. (2021) explored relationships between the social distancing measures introduced in Italy during the first pandemic wave and the development of anxiety in a group of young people attending university. Higher education is a stage in life where people can become more exposed to the risk of developing anxiety and depression due to increased uncertainty about the future. The authors demonstrated that anxiety and related disorders have been negatively affected by the lockdown, though with different intensity depending on the students’ personal features or their housing arrangements. Many of the selected variables proved to have a significant impact on the students’ mental health, some of them increasing the risk of being in a pathological level of anxiety or increasing anxiety level during the lockdown. For example, being a woman proved to be one of the most important factors exposing the student to higher risks of developing serious anxiety and to worsen in case of crisis, emphasizing the increased sense of uncertainty that affects women compared to their pair. The same happened, though at a lower level, to students receiving an income-based scholarships whose uncertainty was also fuelled by the family's limited economic resources. Even changing homes can cause greater uncertainty, while availability of outdoor space resulted to be an asset for students – as well as all the others, actually—to cope with the anxiety caused by being forced to stay in confined spaces for too long. Finally, it is interesting to note the protective action against anxiety that was enacted by the participants’ status as students of a medicine faculty. Seemingly, the great emphasis placed on the importance of this area of study and research during the long “winter” of the pandemic first wave has provided a strong motivational boost for these students eventually enabling them able to reduce their anxiety level.

## Conclusions

### The COVID-19 pandemic has represented for all countries worldwide an unimaginable tragedy

Even industrialised countries proved to be dramatically unprepared, not only for the mitigation of its direct consequences but also, perhaps mainly, for its indirect consequences that pervasively frightened our societies as a whole. All this, despite the investment devoted over the last two decades to the development of pandemic preparedness plans following the numerous alerts that have appeared, ranging from the scare of the avian flu at the end of the previous millennium, to the SARS outbreak in 2003, to the 2009 influenza pandemic, to the MERS and Zika alerts (Horton, 2021; Lupton & Willis, 2021).

Surely, as Richard Horton (2021) was pointing out, this pandemic has destroyed the false feeling of omnipotence that permeated our societies; rather it laid bare the deep vulnerability of our highly interconnected communities at any level and scale, and in any sector, ranging from public health systems, to the economy and to the social tissue. These factors unavoidably put most of the costs of the tragedy on the more frail and more vulnerable people and population groups.

A key question is then whether we will be able to seriously learn, by acquiring full awareness, of the overall dimensions of this tragedy. This will surely represent a task, and a challenge, for governments, policy makers, and for political, economic and education institutions that will have to start rethinking humbly not only their own work, but also their duties towards our communities. With a focus on re-building widespread solidarity as a pre-condition for an equitable and resilient society. This is especially true in view of the future challenges we are called to face, first of all the battle to mitigate the expected effects of climate change. Even science will not be immune and will have to humbly start rethinking its role in our societies, starting from the role of scientific communication, whose disordered and disproportionate presence in the media played a role in the information jam surrounding this pandemic (Bursztyn et al., 2020). We hope that the collection of papers in this Thematic series represent a useful contribution to this process.

## Data Availability

No data has been used in this Editorial.
